# Circulating cell-free mitochondrial DNA levels and glucocorticoid sensitivity in a cohort of male veterans with and without combat-related PTSD

**DOI:** 10.1038/s41398-023-02721-x

**Published:** 2024-01-10

**Authors:** Zachary N. Blalock, Gwyneth W. Y Wu, Daniel Lindqvist, Caroline Trumpff, Janine D. Flory, Jue Lin, Victor I. Reus, Ryan Rampersaud, Rasha Hammamieh, Aarti Gautam, SBPBC, Kerry J. Ressler, Ruoting Yang, Seid Muhie, Bernie J. Daigle, Linda M. Bierer, Leroy Hood, Kai Wang, Inyoul Lee, Kelsey R. Dean, Pramod R. Somvanshi, Francis J. Doyle, Charles R. Marmar, Marti Jett, Rachel Yehuda, Owen M. Wolkowitz, Synthia H. Mellon

**Affiliations:** 1 Department of Psychiatry and Behavioral Sciences and Weill Institute for Neurosciences, University of California, San Francisco, CA USA; 2 Unit for Biological and Precision Psychiatry, Department of Clinical Sciences Lund, Lund University, Lund, Sweden; 3 Department of Psychiatry, Division of Behavioral Medicine, Columbia University Medical Center, New York, USA; 4 James J. Peters VA Medical Center, Bronx, NY USA; 5 Icahn School of Medicine at Mount Sinai, New York, NY USA; 6 Department of Biochemistry and Biophysics, University of California, San Francisco, CA USA; 7 Integrative Systems Biology, US Army Medical Research and Materiel Command, USACEHR, Fort Detrick, Frederick, MD USA; 8 Harvard John A. Paulson School of Engineering and Applied Sciences, Harvard University, Cambridge, MA USA; 9 Department of Psychiatry, New York University Grossman School of Medicine, New York, NY USA; 10 Department of Obstetrics, Gynecology, & Reproductive Sciences, University of California, San Francisco, CA USA; 11 Department of Psychiatry, Harvard Medical School and McLean Hospital, Belmont, MA USA; 12 Departments of Biological Sciences and Computer Science, The University of Memphis, Memphis, TN USA; 13 Institute for Systems Biology, Seattle, WA USA

**Keywords:** Diagnostic markers, Physiology, Psychiatric disorders, Human behaviour

## Abstract

Circulating cell-free mitochondrial DNA (ccf-mtDNA) is a biomarker of cellular injury or cellular stress and is a potential novel biomarker of psychological stress and of various brain, somatic, and psychiatric disorders. No studies have yet analyzed ccf-mtDNA levels in post-traumatic stress disorder (PTSD), despite evidence of mitochondrial dysfunction in this condition. In the current study, we compared plasma ccf-mtDNA levels in combat trauma-exposed male veterans with PTSD (*n* = 111) with those who did not develop PTSD (*n* = 121) and also investigated the relationship between ccf mt-DNA levels and glucocorticoid sensitivity. In unadjusted analyses, ccf-mtDNA levels did not differ significantly between the PTSD and non-PTSD groups (*t* = 1.312, *p* = 0.191, Cohen’s d = 0.172). In a sensitivity analysis excluding participants with diabetes and those using antidepressant medication and controlling for age, the PTSD group had lower ccf-mtDNA levels than did the non-PTSD group (F(1, 179) = 5.971, *p* = 0.016, partial *η*^2^ = 0.033). Across the entire sample, ccf-mtDNA levels were negatively correlated with post-dexamethasone adrenocorticotropic hormone (ACTH) decline (*r* = −0.171, *p* = 0.020) and cortisol decline (*r* = −0.149, *p* = 0.034) (viz., greater ACTH and cortisol suppression was associated with lower ccf-mtDNA levels) both with and without controlling for age, antidepressant status and diabetes status. Ccf-mtDNA levels were also significantly positively associated with IC_50-DEX_ (the concentration of dexamethasone at which 50% of lysozyme activity is inhibited), a measure of lymphocyte glucocorticoid sensitivity, after controlling for age, antidepressant status, and diabetes status (*β* = 0.142, *p* = 0.038), suggesting that increased lymphocyte glucocorticoid sensitivity is associated with lower ccf-mtDNA levels. Although no overall group differences were found in unadjusted analyses, excluding subjects with diabetes and those taking antidepressants, which may affect ccf-mtDNA levels, as well as controlling for age, revealed decreased ccf-mtDNA levels in PTSD. In both adjusted and unadjusted analyses, low ccf-mtDNA levels were associated with relatively increased glucocorticoid sensitivity, often reported in PTSD, suggesting a link between mitochondrial and glucocorticoid-related abnormalities in PTSD.

## Introduction

Post-traumatic stress disorder (PTSD) is a debilitating psychiatric disorder that occurs in some individuals as a result of exposure to a severe traumatic event. Presently, it is estimated to affect 1.1% of the global population within any 12-month period [1]. PTSD prevalence is higher among those with combat trauma exposure [2], and prevalence among Operation Iraqi Freedom (OIF) and Operation Enduring Freedom (OEF) veterans has been estimated to be 15.8% [3]. Uncovering biomarkers clearly associated with PTSD or a subset of its symptoms could aid in more effective diagnosis, prognosis, and treatment as well as possibly refining our understanding of PTSD pathophysiology. Circulating cell-free mitochondrial DNA (ccf-mtDNA) is derived and released from cells into the systemic circulation following cellular injury or cellular stress. Its release can occur passively via different forms of cell death or actively from living cells through more regulated processes, which are not yet fully understood [4]. Ccf-mtDNA has received increased attention as a biomarker for a variety of somatic pathologies. Increased blood ccf-mtDNA levels have been associated with conditions including diabetes [5–9], heart disease [5, 6], and inflammatory diseases [10], as well as with aging [8, 11, 12] and certain cancers [13–15]. Conversely, decreased ccf-mtDNA levels in cerebrospinal fluid (CSF) have been observed in some neurodegenerative conditions including some types of Parkinson’s disease and Alzheimer’s disease [16–18], where they have been suggested to possibly reflect mitochondrial loss in early stages of disease progression [18].

Although ccf-mtDNA has not been specifically investigated in PTSD, some studies have explored the relationship between ccf-mtDNA levels and other psychiatric disorders. Elevated plasma ccf-mtDNA levels have been associated with late-life depression (LLD) [19], with LLD combined with frailty [20], and with MDD in unmedicated individuals [21]. Notably, antidepressant administration in individuals with depression altered plasma ccf-mtDNA levels differentially in antidepressant treatment responders vs. non-responders [21], being associated with increased ccf-mtDNA levels in non-responders only. Lindqvist et al. [22] found that plasma ccf-mtDNA was significantly increased in individuals who had recently attempted suicide compared to controls, and ccf-mtDNA levels were positively correlated with post-dexamethasone cortisol levels, suggesting an inverse relationship with glucocorticoid sensitivity in these individuals. A positive association was also found between acute psychosocial stress and ccf-mtDNA levels in plasma [23] and serum [24]. On the other hand, significantly lower plasma ccf-mtDNA has been reported in unmedicated [25] and medicated [26] individuals with MDD, in individuals with social anxiety disorder [27], and in individuals with bipolar disorder [25]. In addition, no significant differences in serum ccf-mtDNA levels relative to controls were found in studies of females with MDD [28] and in individuals with bipolar disorder [29, 30], and a recent meta-analysis of ccf-mtDNA levels and brain disease that included studies of MDD, suicidality, bipolar disorder, and schizophrenia did not find a significant overall difference between psychiatric cases and controls [31]. The reasons for the discrepant results thus far are unclear but raise the possibility that individual factors apart from the psychiatric diagnoses per se (e.g. comorbid psychiatric or medical conditions or medications, comorbid metabolic/inflammatory imbalances, or associated hypothalamic-pituitary-adrenal [HPA] axis alterations) may confound certain results [26].

A number of conditions closely associated with PTSD, including suicidal ideation [32–34], elevated inflammation [35], and metabolic disorders such as type 2 diabetes [36], were found to be associated with elevated ccf-mtDNA, although these have not been specifically studied in individuals with PTSD. However, increased glucocorticoid sensitivity has been reported in multiple studies of PTSD [2, 37, 38], and based on the positive associations reported between ccf-mtDNA and glucocorticoid levels following physical stress [23] and between post-dexamethasone suppression test (DST) cortisol levels and ccf-mtDNA levels in individuals who had attempted suicide [22], it seems plausible that increased glucocorticoid sensitivity in individuals with PTSD could be associated with lower ccf-mtDNA levels.

The main aim of this study was to test our hypothesis that ccf-mtDNA levels would be altered in individuals with PTSD compared to the PTSD negative controls, although we did not specify the direction of this alteration. We compared plasma ccf-mtDNA levels between individuals with and without PTSD in a large, well-characterized group of male veterans, all of whom had been exposed to combat trauma. We also conducted a sensitivity analysis to control for the influence of diabetes status, antidepressant medication, and age. Moreover, we analyzed ccf-mtDNA levels in relation to glucocorticoid sensitivity, antidepressant medication, and psychometric scores.

## Methods

### Study participants

Male combat trauma-exposed veterans who had served in active duty in Operation Iraqi Freedom (OIF) or Operation Enduring Freedom (OEF) were recruited by New York University (NYU), the Icahn School of Medicine at Mount Sinai (ISMMS), and the James J. Peters Veterans Administration Medical Center (JJPVAMC). Detailed descriptions of the recruitment procedure and exclusion criteria for study participants can be found in prior publications [39–41]. Briefly, inclusion criteria for the PTSD group were age 20–60 years and current diagnosis of war zone-related PTSD based on the Structured Clinical Interview for DSM-IV-*TR* [42], which was the DSM version in use at the time of this study, and the Clinician-Administered PTSD Scale (CAPS) [43, 44]. Current PTSD diagnosis was determined by the *DSM-IV-TR* criteria. Combat trauma-exposed non-PTSD controls were also included if aged between 20 and 60, had previously served in war zones, were free from a lifetime history of PTSD, and had a current CAPS score less than or equal to 20. All participants (PTSD-positive and PTSD-negative) met DSM-IV diagnostic criterion A of the PTSD diagnosis for combat trauma exposure, and the time since the index trauma was 6 years (±2.8 years) on average. The exclusion criteria for the study included the following: prominent suicidal or homicidal ideation; a history of alcohol dependence within the past eight months or of substance abuse or dependence other than nicotine within the past year; a lifetime history of any psychiatric disorder with psychotic features, bipolar disorder, obsessive-compulsive disorder, any neurologic disorder, any systemic illness affecting central nervous system (CNS) function, or a moderate or severe traumatic brain injury (TBI) based on the Ohio State University TBI Identification Method––Short Form [45]; and a history of anemia or recent blood donation in the past 2 months. In addition, individuals who were exposed to ongoing trauma or had been exposed to a traumatic event within the past three months and who were not stable for at least two months on psychiatric medication, anticonvulsants, antihypertensive medication, or sympathomimetic medication were excluded. Individuals with medical conditions that may affect systemic inflammation were also excluded. In total, 232 participants had available ccf-mtDNA data, and they were grouped into those with (*n* = 111) and those without (*n* = 121) PTSD. A power analysis using G*Power version 3.1.9.7 showed our sample size achieved 80% power for detecting a medium effect size of 0.4. The study was approved by the Institutional Review Board of the UCSF School of Medicine, the ISMMS, the JJPVAMC, the NYU Grossman School of Medicine, and the US Army Medical Research and Materiel Command. All participants provided written informed consent before participation in the study. The study was conducted in accordance with the Declaration of Helsinki (1989).

### Evaluation of psychiatric symptoms

PTSD status of all participants was determined by doctoral-level psychologists using the Structured Clinical Interview for DSM-IV Axis I Disorders [46] and the Clinician Administered PTSD Scale (CAPS) [43]. A review panel including senior experienced clinicians reviewed all diagnoses. Participants also self-reported their PTSD symptoms, depression symptoms, and perceived psychological stress via the PTSD Checklist for DSM-IV (PCL) [47], the Beck Depression Inventory-II (BDI-II) [48], and the Perceived Stress Scale-10 [49], respectively.

### Blood collection

Blood was drawn by venipuncture in the morning between 8am to 8:30am after a night of fasting in all participants. For plasma analyses, blood was collected into tubes containing EDTA. After being inverted 8–10 times, tubes were put on ice for a maximum of 30 minutes and then spun at 1100 x g for 15 minutes at 4 °C. Plasma was removed and divided into 500 μL aliquots, which were stored at −80 °C for later analysis.

### Ccf-mtDNA purification and quantification

Plasma samples were stored at −80 °C after collection, thawed, and spun at 10,000 × *g* for 10 min to remove cellular debris. DNA was isolated from 200 μL of plasma using the QIAamp DNA Blood Mini Kit (Cat #51106, Qiagen, Valencia, CA, USA) according to the manufacturer’s instructions. The isolated DNA was eluted in 60 μL AE buffer and stored at −80 °C before being assayed. The quantitative analysis of cell free-mtDNA was performed using quantitative real-time polymerase chain reaction (qPCR) by amplifying a 161 bp product from the NADH:Ubiquinone Oxidoreductase Core Subunit 2 (MT-ND2) gene. Each 10 μL reaction was comprised of 5 μL QuantiFast SYBR Green PCR Kit (Qiagen, Valencia, CA, USA), 0.5 μL of each primer, and 2.5 μL of extracted DNA. Each reaction was run in triplicate on an LC480 (LightCycler, Roche, Mannheim, Germany) using the following program: Initial denaturation at 95 °C for ten minutes, and then 45 cycles of the following: melting at 95 °C for 10 s, annealing at 65 °C for 10 s, and extension at 72 °C for 11 s. Finally, a melting curve analysis measured fluorescence continuously from 60 °C to 97°. The forward primer sequence was 5′-CACACTCATCACAGCGCTAA-3′, and the reverse primer sequence was 5′-GGATTATGGATGCGGTTGCT-3′.

A 7-point, 4-fold serial dilution of genomic DNA (Roche Life Science, Indianapolis, Indiana, United States) was created as the standard curve to quantify the mitochondrial DNA copy number relative to the genomic DNA using the Roche480’s absolute quantification maximum second derivative method. The highest concentration of the standard curve was 20 ng/μl. In order to convert the measured relative copy numbers to absolute copy numbers, the same primers were used to amplify a PCR product from the human genomic DNA (Roche Life Science, Indianapolis, Indiana, United States, cat # 1169111200). The PCR product was purified using the QIAquick PCR Purification Kit (Qiagen, Valencia, CA, USA) protocol. A PicoGreen Assay (Cat ThermoFisher Scientific Inc., Waltham, Massachusetts, USA) was conducted to obtain an accurate concentration of the product. The relative concentration of the PCR product was measured using the human genomic DNA as the reference standard, and the absolute mtDNA copy number concentration of each sample was then converted accordingly. The copy number of the PCR product was calculated first by dividing the mass by its molecular weight, calculated based on its unique sequence (http://www.bioinformatics.org/sms2/dna_mw.html), and multiplied by Avogadro’s constant. The final values are expressed as copy numbers/μL of plasma. The average inter-assay CV from 24 randomly picked samples was 3.8 ± 1.9%.

### Clinical labs

Clinical labs were conducted by a CLIA-certified lab.

### Dexamethasone suppression test and neuroendocrine assays

The dexamethasone suppression test (DST) was conducted as previously described (Somvanshi et al., 2020) to measure the negative feedback response of the HPA axis. Dexamethasone was administered orally at a dose of 0.5 mg at 11:00 pm the night before the second blood draw, which took place at 8:00 am the following day. Plasma cortisol was assayed with a Cortisol ELISA Kit (IBL-America, Minneapolis, MN), with intra- and inter-assay coefficients of variation of 5.3% and 9.8%, respectively. Assay sensitivity was 2.5 ng/mL. Plasma ACTH was assayed using an ACTH ELISA kit (ALPCO Diagnostics, Windham, NH), and the intra- and inter-assay coefficients of variation were 5.0% and 8.7%, respectively. Assay sensitivity was 0.5 pg/mL. Individuals with no dexamethasone values for day 2 (PTSD negative: *n* = 15; PTSD positive: *n* = 11) were excluded from analyses of post-dexamethasone measures. Declines of cortisol and ACTH from day 1 to day 2 were used as measures of dexamethasone suppression.

### Dexamethasone-induced lysozyme suppression (IC_50-DEX_) assay

This test, which is used to determine the concentration of dexamethasone at which 50% of lysozyme activity is inhibited (IC_50-DEX_) in peripheral blood mononuclear cells (PBMCs), was performed as previously described in detail [50, 51]. Briefly, PBMCs were isolated on the day of blood collection via density gradient centrifugation using Ficoll-Paque media (Pharmacia), and the IC_50-DEX_ assay was performed the same day. The turbidimetric method was used to determine lysozyme inhibition in cells that were incubated with *Micrococcus lysodeikticus* (Sigma) in dexamethasone concentrations of 0, .5, 1, 2.5, 5, 10, 50, and 100 nmol/L. IC_50-DEX_ refers to the concentration of dexamethasone at which a 50% reduction in lysozyme activity was observed. Accordingly, higher IC_50-DEX_ values correspond to lower glucocorticoid sensitivity. The intra-assay coefficient of variation was 6.9%, and the inter-assay coefficient of variation was 9.8%.

### Statistical analysis

Statistical analyses were performed using the Statistical Package for the Social Sciences (SPSS), version 28.0 (SPSS Inc., Chicago, IL, USA). Variables were assessed for normality, and Blom transformation [52] was applied to those with non-normal distribution. Descriptive statistics were calculated for socio-demographic characteristics and biological markers. Continuous variables were summarized with means ± standard deviations (SD) and compared with independent t-tests, and categorical variables were summarized with frequencies and compared with chi-squared tests. To analyze group differences in ccf-mtDNA levels between PTSD-positive and PTSD-negative subjects, a two-tailed Student’s t-test was used.

We then conducted a sensitivity analysis and ANCOVA to control for the confounding effects of age, diabetes status, and antidepressant use. These variables were selected based on significant associations reported in the literature between ccf-mtDNA levels and diabetes [5–9] and age [8, 11, 12], and our previous findings in a cohort of MDD subjects suggesting higher ccf-mtDNA levels in individuals with MDD who did not respond to SSRIs relative to responders [21]. Diabetes was defined as having hemoglobin A1c (HbA1c) values higher than 6.5% (PTSD, *n* = 6; control, *n* = 3). We excluded participants with diabetes and those who were taking antidepressants and performed an ANCOVA model with age as a covariate.

For analysis of the relationship between ccf-mtDNA levels and glucocorticoid-related measures, two-tailed Pearson correlations were performed first between ccf-mtDNA levels and continuous variables. We then conducted multiple regression analysis including antidepressant use, age, and diabetes status as covariates.

A two-tailed t-test was used to analyze differences in ccf-mtDNA levels between antidepressant users and non-users within the PTSD group, and ANCOVA was used to control for age and diabetes status.

Two-tailed Pearson correlations were used for analyses of the association between ccf-mtDNA levels and age, body mass index (BMI), HbA1c, and psychometric scores. For all continuous variables analyzed other than age, multiple regression analysis was performed with age, diabetes status, and antidepressant use as covariates.

## Results

### Characteristics of subjects and between-group differences

Demographic and clinical characteristics of our sample are shown in Table 1. The PTSD and control groups did not differ significantly in terms of age. There were significant differences in BMI (*t* = −2.296, *p* = 0.023), ethnicity (*χ*^2^ = 12.423, *p* = 0.029), smoking status (*χ*^2^ = 17.125, *p* < 0.001), and years of education (*t* = 3.296, *p* < 0.001). Greater antidepressant use (*χ*^2^ = 24.000, *p* < 0.001) was found in PTSD positive subjects.

**Table 1:** Demographic and clinical characteristics.

	Total (*n* = 232)	Control (*n* = 121)	PTSD (*n* = 111)	p value^d^
Age (years, mean ± s.d.)	33.44 ± 8.55	33.08 ± 8.61	33.84 ± 8.51	0.503
Education (years, mean ± s.d.)	14.45 ± 2.11	14.96 ± 2.15	13.9 ± 1.94	<0.001
BMI (mean ± s.d.)	28.93 ± 5.00	28.19 ± 4.40	29.72 ± 5.48	0.023
Race/ethnicity				0.029
Non-Hispanic Asian, *n* (%)	15 (6.5)	11 (9.1)	4 (3.6)	–
Non-Hispanic Black, *n* (%)	52 (22.4)	26 (21.5)	26 (23.4)	–
Non-Hispanic Native American, *n* (%)	2 (0.9)	2 (1.7)	0 (0)	–
Non-Hispanic Other, *n* (%)	7 (3.0)	4 (3.3)	3 (2.7)	–
Non-Hispanic White, *n* (%)	75 (32.3)	46 (38.0)	29 (26.1)	–
Hispanic, *n* (%)	81 (34.9)	32 (26.4)	49 (44.1)	–
Antidepressant users, *n* (%)^a^	35 (15.1)	5 (4.1)	30 (27.0)	<0.001
Smoking Status^b^				<0.001
Not at all, *n* (%)	157 (80.5)	94 (88.7)	63 (70.8)	–
Some days, *n* (%)	15 (7.7)	8 (7.5)	7 (7.9)	–
Every day, *n* (%)	23 (11.8)	4 (3.8)	19 (21.3)	–
Current CAPS total score (mean ± s.d.)	34.88 ± 34.90	3.83 ± 5.09	68.72 ± 17.69	<0.001
HbA1c (%, mean ± s.d.)	5.40 ± 0.75	5.33 ± 0.47	5.47 ± 0.99	0.766
Participants with Diabetes (HbA1c > 6.5%)^c^	9	3	6	0.198

*BMI* body mass index, *HbA1c* hemoglobin A1c, *CAPS* Clinician-Administered PTSD Scale.

^a^Data on antidepressant use was not available for 1 participant in the PTSD positive group.

^b^Data on smoking status was not available for 37 participants (PTSD negative: *n* = 15; PTSD positive: *n* = 22).

^c^Data on HbA1c was not availalble for 9 participants in the PTSD positive group.

^d^For continuous variables, a t-test was used. For categorical variables, a *χ*^2^ test was used.

### PTSD status and ccf-mtDNA

**Fig. 1:**
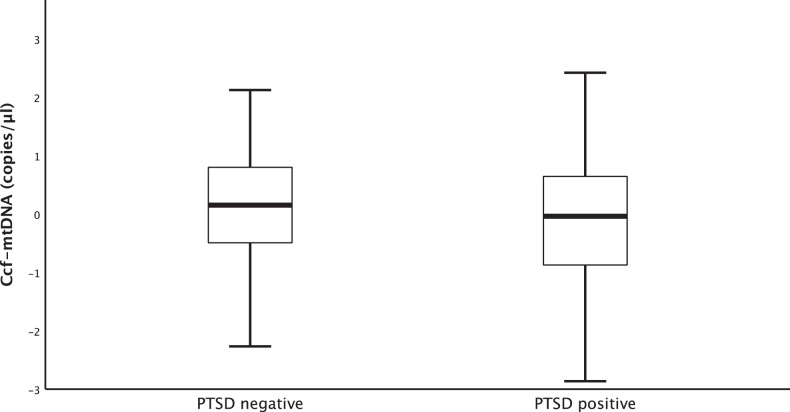
Associations between PTSD and ccf-mtDNA levels. After adjusting for age, diabetes status, and antidepressant medication, ccf-mtDNA levels were significantly lower in the PTSD group (PTSD negative: *M* = 0.136, SD = 0.085, 95% CI [−0.032, 0.304]; PTSD positive: *M* = −0.163, SD = 0.933, 95% CI [−0.368, 0.043]; *t* = 2.250, df = 220, *p* = 0.025, Cohen’s d = 0.303). Data represent residual values after adjusting for age, diabetes status, and antidepressant use.

A between-group t-test did not show a significant difference in ccf-mtDNA levels between the PTSD and control groups (PTSD negative: *M* = 0.082 (standardized value), SD = 0.934, 95% CI [−0.086, 0.250]; PTSD positive: *M* = −0.089 (standardized value), SD = 1.053, 95% CI [−0.287, 0.109]; *t* = 1.312, df = 230, *p* = 0.191, Cohen’s *d* = 0.172). After excluding participants with diabetes and those who were taking antidepressants and controlling for age in an ANCOVA model, however, we found significantly lower ccf-mtDNA levels in the PTSD group (F(1, 179) = 5.971, *p* = 0.016, partial *η*^2^ = 0.033; PTSD negative: estimated marginal mean = 0.055, 95% CI [−0.121, 0.231]; PTSD positive: estimated marginal mean = −0.303, 95% CI [−0.532, −0.074]) (Fig. 1). Excluding those with a current MDD diagnosis instead of antidepressant use did not result in a significant between-group difference (*F*(1, 159) = 2.537, *p* = 0.113, partial *η*^2^ = 0.016; PTSD negative: estimated marginal mean = 0.075, 95% CI [−0.103, 0.254]; PTSD positive: estimated marginal mean = −0.191, 95% CI [−0.468, 0.086]), implicating antidepressant medication rather than the diagnosis of MDD.

### Ccf-mtDNA and glucocorticoid signaling

**Fig. 2:**
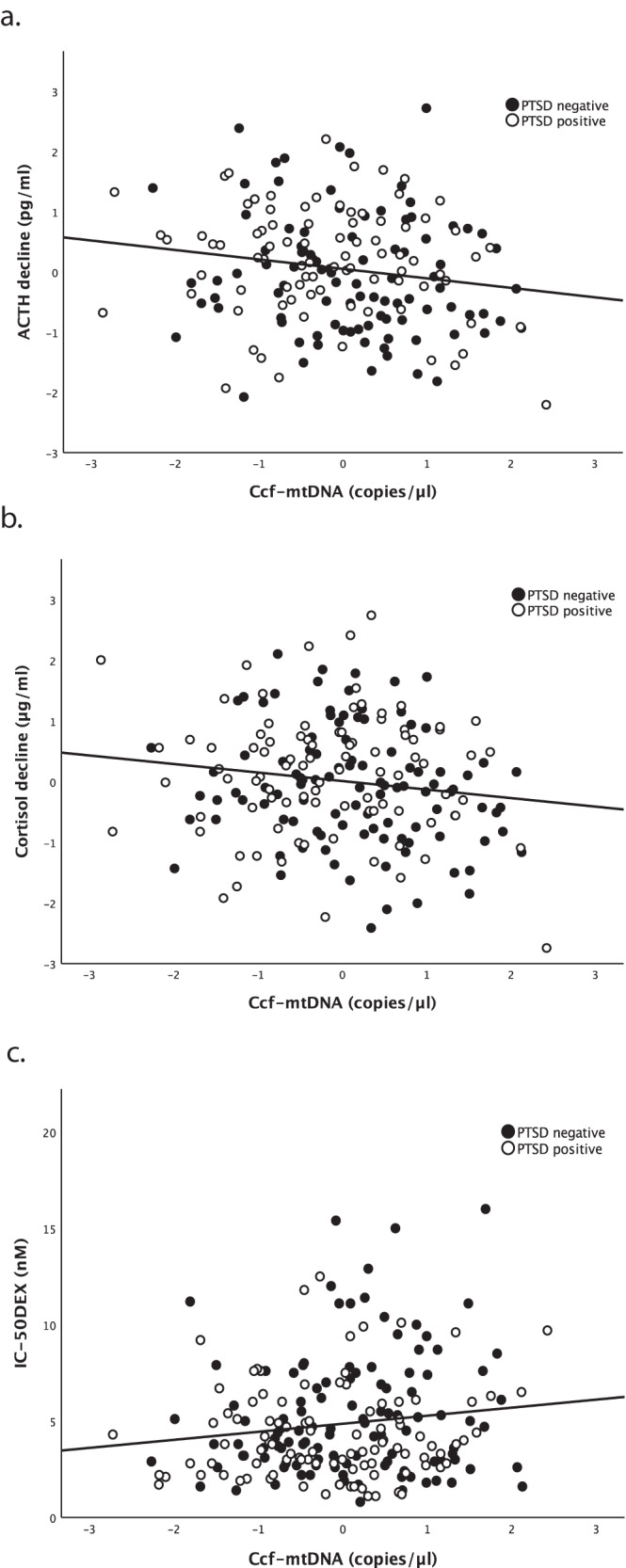
Correlations between ccf-mtDNA levels and glucocorticoid sensitivity after adjusting for age, diabetes status, and antidepressant use. Data represent residual values after adjusting for age, diabetes status, and antidepressant use. **a** Ccf-mtDNA levels were negatively correlated with ACTH decline across the entire group (*r* = −0.168, *p* = 0.024). **b** Ccf-mtDNA levels were also negatively correlated with cortisol decline across the entire group (*r* = −0.143, *p* = 0.045). **c** IC_50-DEX_, for which lower values indicate higher glucocorticoid sensitivity, was positively correlated with ccf-mtDNA levels across the entire group (*r* = 0.140, *p* = 0.043).

Our analyses of DST measures showed that, across the entire group, but not within each individual group, ccf-mtDNA levels were negatively correlated with post-dexamethasone ACTH decline (*r* = −0.171, *p* = 0.020) and cortisol decline (*r* = −0.149, *p* = 0.034; Table 2). Specifically, lower ccf-mtDNA levels were associated with larger dexamethasone-associated decreases in ACTH and cortisol, indicating greater glucocorticoid sensitivity in the HPA axis. The correlations for ACTH decline and ccf-mtDNA levels and for cortisol decline and ccf-mtDNA levels remained significant when controlling for age, diabetes status, and antidepressant use (ACTH decline: *β* = −0.170, *p* = 0.020; cortisol decline: *β* = −0.143, *p* = 0.043) (Table 3 and Fig. 2a, b).

**Table 2:** Correlations of continuous variables with ccf-mtDNA and group comparisons for each variable.

Variable	Entire Group		PTSD-		PTSD+		PTSD-	PTSD+			
	Pearson’s *r*	*p* value	Pearson’s *r*	*p* value	Pearson’s *r*	*p* value	mean ± s.d.	mean ± s.d.	*t*	df	*p* value
age (years)	0.187	0.004	0.181	0.047	0.204	0.032	33.08 ± 8.61	33.84 ± 8.51	−0.671	230	0.503
BMI	0.031	0.639	0.035	0.706	0.053	0.583	28.19 ± 4.40	29.72 ± 5.48	−2.296	206.997	0.023
HbA1c (%)^a^	0.245	<0.001	0.248	0.006	0.249	0.012	5.33 ± 0.47	5.47 ± 0.99	−0.299	221	0.766
pre-dex cortisol (µg/ml)^a^	−0.038	0.567	−0.058	0.525	−0.015	0.881	14.13 ± 6.01	14.71 ± 7.47	−0.32	228	0.749
post-dex cortisol (µg/ml)^a^	0.064	0.358	0.001	0.99	0.117	0.248	3.77 ± 4.36	3.76 ± 6.38	0.557	203	0.578
pre-dex ACTH (pg/ml)^a^	−0.096	0.171	−0.081	0.409	−0.102	0.311	36.81 ± 26.95	38.21 ± 20.65	−1.508	194.866	0.133
post-dex ACTH (pg/ml)^a^	0.086	0.234	0.127	0.208	0.039	0.71	14.76 ± 11.69	13.60 ± 10.45	0.897	184	0.371
ACTH decline (pg/ml)^a^	−0.171	0.02	−0.146	0.157	−0.181	0.088	22.26 ± 26.91	24.50 ± 19.11	−1.686	183	0.094
cortisol decline (µg/ml)^a^	−0.149	0.034	−0.183	0.061	−0.106	0.297	10.21 ± 6.17	11.24 ± 7.20	−1.188	201	0.236
IC_50-DEX_ (nM)	0.131	0.051	0.143	0.131	0.102	0.29	5.29 ± 3.24	4.33 ± 2.43	2.516	219	0.013
Time since worst event (months)	0.074	0.264	0.184	0.043	−0.012	0.901	65.54 ± 35.20	77.77 ± 30.25	−2.827	230	0.005
BDI2 total	−0.107	0.111	−0.157	0.093	−0.024	0.809	5.94 ± 6.52	24.05 ± 10.96	−14.782	167.88	<0.001
PCL total	−0.121	0.07	−0.168	0.069	−0.021	0.832	25.62 ± 9.27	59.54 ± 13.03	−22.224	187.485	<0.001
CAPS total (current)	−0.107	0.105	−0.221	0.015	−0.042	0.659	3.83 ± 5.09	68.72 ± 17.69	−37.252	126.702	<0.001
CAPS criterion B (current)	−0.081	0.217	−0.059	0.523	−0.014	0.885	0.29 ± 0.98	16.99 ± 7.46	−23.39	113.469	<0.001
CAPS criterion C (current)	−0.085	0.197	−0.138	0.132	0.022	0.816	0.98 ± 2.55	27.35 ± 7.80	−34.001	131.55	<0.001
CAPS criterion D (current)	−0.141	0.031	−0.192	0.035	−0.131	0.172	2.56 ± 3.73	24.38 ± 6.28	−31.796	175.769	<0.001
CAPS total (lifetime)	−0.105	0.111	−0.139	0.127	−0.05	0.603	8.90 ± 7.88	89.22 ± 18.97	−41.438	144.249	<0.001
Perceived Stress Scale	−0.077	0.258	−0.06	0.519	−0.013	0.897	1.85 ± 0.61	2.98 ± 068	−13.039	217	<0.001

*PTSD* post-traumatic stress disorder, *BMI* body mass index, *HbA1c* hemoglobin A1c, *ACTH* adrenocorticotropic hormone, *IC*_*50-DEX*_ the concentration of dexamethasone at which 50% of lysozyme activity is inhibited, *BDI2* Beck Depression Inventory 2, *PCL* PTSD checklist, *CAPS* Clinician-Administered PTSD Scale.

^a^Standardized values were used for correlations and *t*-tests, but means represent raw values.

**Table 3:** Associations with ccf-mtDNA controlling for age, diabetes status, and antidepressant use.

Variable	Entire group		PTSD-		PTSD+	
	*β*	*p* value	*β*	*p* value	*β*	*p* value
BMI	−0.021	0.767	0	0.998	−0.008	0.941
HbA1c	0.203	0.006	0.21	0.029	0.285	0.084
Pre-dex cortisol (µg/ml)^a^	−0.033	0.614	−0.06	0.511	0.011	0.91
Post-dex cortisol (µg/ml)^a^	0.05	0.475	0.008	0.931	0.082	0.423
Pre-dex ACTH (pg/ml)^a^	−0.116	0.097	−0.091	0.358	−0.113	0.28
Post-dex ACTH (pg/ml)^a^	0.059	0.423	0.123	0.245	−0.005	0.962
ACTH decline (pg/ml)^a^	−0.170	0.02	−0.143	0.168	−0.163	0.132
Cortisol decline (µg/ml)^a^	−0.143	0.043	−0.18	0.069	−0.069	0.506
IC_50-DEX_ (nM)	0.142	0.038	0.135	0.159	0.099	0.329
Time since worst event (months)	−0.051	0.449	0.147	0.119	0.012	0.904
BDI2 score	−0.184	0.011	−0.195	0.039	−0.019	0.854
pcl score	−0.187	0.008	−0.177	0.055	0.019	0.847
CAPS total score (current)	−0.192	0.007	−0.238	0.009	−0.062	0.533
CAPS B (current)	−0.168	0.018	−0.117	0.21	−0.059	0.552
CAPS C (current)	−0.155	0.03	−0.118	0.197	0.033	0.742
CAPS D (current)	−0.226	0.001	−0.216	0.018	−0.141	0.155
CAPS total score (lifetime)	−0.194	0.007	−0.165	0.079	−0.075	0.453
Perceived Stress Scale	−0.118	0.094	−0.069	0.468	0.031	0.758

*PTSD* post-traumatic stress disorder, *BMI* body mass index, *HbA1c* hemoglobin A1c, *ACTH* adrenocorticotropic hormone, *IC*_*50-DEX*_ the concentration of dexamethasone at which 50% of lysozyme activity is inhibited, *BDI2* Beck Depression Inventory 2, *PCL* PTSD Checklist, *CAPS* Clinician-Administered PTSD Scale.

^a^Standardized values were used.

IC_50-DEX_, a measure of glucocorticoid sensitivity in PBMCs, was significantly lower in PTSD subjects relative to controls (PTSD negative: *M* = 5.295, SD = 3.239, 95% CI [4.688, 5.901]; PTSD positive: *M* = 4.328, SD = 2.429, 95% CI [3.866, 4.789]; *t* = 2.516, df = 205.743, *p* = 0.013, Cohen’s d = 0.337) (Table 2), indicating greater glucocorticoid sensitivity. Though IC_50-DEX_ was not significantly associated with ccf-mtDNA levels in unadjusted analyses (*r* = 0.131, *p* = 0.51) (Table 2), after controlling for age, diabetes status, and antidepressant use, IC_50-DEX_ was significantly positively associated with ccf-mtDNA levels across the entire group (*β* = 0.142, *p* = 0.038), but not within each individual group (Table 3 and Fig. 2c).

### Ccf-mtDNA and antidepressant use in PTSD subjects

Based on our ANCOVA results, we also explored the relationship between antidepressant use and ccf-mtDNA within the PTSD group. Antidepressant users had higher ccf-mtDNA levels, although the difference missed statistical significance (PTSD only: *M* = −0.199, SD = 1.056, 95% CI [−0.434, 0.036]; PTSD + Antidepressants: *M* = 0.211, SD = 1.020, 95% CI [−0.170, 0.592]; *t* = −1.832, *p* = 0.070, Cohen’s *d* = −0.392). The difference was significant (*F*(1, 100) = 4.082, *p* = 0.046; PTSD only: estimated marginal mean = −0.270, 95% CI [−0.513, −0.027]; PTSD + Antidepressants: estimated marginal mean = 0.200, 95% CI [−0.187, 0.586]), however, when controlling for age and diabetes status. Ccf-mtDNA levels did not differ significantly between PTSD subjects with concurrent MDD and those without concurrent MDD (PTSD only: *M* = −0.018, SD = 1.137, 95% CI [−0.326, 0.289]; PTSD + MDD: *M* = −0.159, SD = 0.969, 95% CI [−0.418, 0.101]; *t* = 0.699, *p* = 0.486, Cohen’s *d* = 0.133), including when controlling for age, diabetes, and antidepressant status (*F*(1, 101) = 0.311, *p* = 0.579; PTSD only: estimated marginal mean = −.076, 95% CI [−.0373, 0.220]; PTSD + MDD: estimated marginal mean = −0.192, 95% CI [−0.478, 0.093]), again implicating antidepressant medication rather than a comorbid MDD diagnosis.

### Exploratory correlation analyses

Exploratory analyses were performed to examine potential associations between ccf-mtDNA levels, age, BMI, HbA1c, and psychometric test scores (Tables 2 and 3). Ccf-mtDNA levels were significantly positively correlated with age across the entire group (*r* = 0.187, *p* = 0.004) and separately within the PTSD negative group (*r* = 0.181, *p* = 0.047) and the PTSD positive group (*r* = 0.204, *p* = 0.032). Ccf-mtDNA levels were also significantly associated with HbA1c across the entire group (*r* = 0.245, *p* < 0.001) and within each group separately (PTSD negative: *r* = 0.248, *p* = 0.006; PTSD positive: *r* = 0.249, *p* = 0.012). BMI was not significantly associated with ccf-mtDNA levels across the entire group or within either group.

Within the PTSD group, there were no significant associations found between ccf-mtDNA and CAPS lifetime score, CAPS criterion B score, CAPS criterion C score, CAPS criterion D score, PCL score, time since worst event, BDI-II score, or PSS score, before and after controlling for age, diabetes status, and antidepressant use (Tables 2 and 3). Within the PTSD negative group, ccf-mtDNA levels were significantly negatively correlated with current CAPS score (*r* = −0.221, *p* = 0.015), CAPS criterion D score (*r* = −0.192, *p* = 0.035), and time since worst event (*r* = 0.184, *p* = 0.043). After controlling for age, diabetes status, and antidepressant use, current CAPS score (*β* = −0.238, *p* = 0.009), CAPS criterion D score (*β* = −0.216, *p* = 0.018), and BDI-II score (*β* = −0.195, *p* = 0.039) were negatively associated with ccf-mtDNA levels within the PTSD negative group. Across the entire group, CAPS criterion D score was negatively correlated with ccf-mtDNA levels (*r* = 0.141, *p* = 0.031). After controlling for age, diabetes status, and antidepressant medication, CAPS current score (*β* = −0.192, *p* = 0.007), CAPS lifetime score (*β* = −0.194, *p* = 0.007), CAPS criterion B score (*β* = −0.168, *p* = 0.018), CAPS criterion C score (*β* = 0.155, *p* = 0.03, CAPS criterion D score (*β* = −0.226, *p* = 0.001), PCL score (*β* = −0.187, *p* = 0.008), and BDI-II score (*β* = −0.184, *p* = 0.011) were all negatively associated with ccf-mtDNA levels across the entire group.

## Discussion

To our knowledge, this is the first study to examine the relationship between ccf-mtDNA levels and PTSD. Ccf-mtDNA levels did not differ between PTSD positive subjects and PTSD negative controls in the unadjusted analysis. After controlling for age, diabetes status, and antidepressant use (all variables that may affect ccf-mtDNA levels), however, we found significantly lower ccf-mtDNA in the PTSD positive group. We also found an association between glucocorticoid sensitivity and ccf-mtDNA levels, such that higher glucocorticoid sensitivity was associated with lower ccf-mtDNA levels. Finally, we replicated previously reported correlations between ccf-mtDNA levels and age [11, 12] and HbA1c [7].

The small number of studies that have examined ccf-mtDNA in psychiatric conditions have been inconclusive [28, 49], and comparing their results is complicated by differences in sample characteristics, exclusion criteria, and ccf-mtDNA purification protocols [4]. Although there have been no prior studies of ccf-mtDNA in PTSD, there have been studies in MDD and in suicidality, which are common PTSD comorbidities and which were associated with elevated plasma ccf-mtDNA in two prior studies by our group, one in unmedicated MDD subjects [21] and another in suicide attempters [22]. In contrast, Fernström et al [26]. reported that current depression and remitted depression were both negatively associated with ccf-mtDNA levels, though over 90% of subjects in that study were on psychotropic medication. Kageyama et al. [25] also reported significantly lower ccf-mtDNA relative to controls in unmedicated MDD subjects. Although the relationship between MDD and ccf-mtDNA remains unclear, because MDD was overrepresented in the PTSD group (*n* = 56) relative to the control group (*n* = 4), it seemed plausible that MDD could have affected the association we found between PTSD and ccf-mtDNA. However, ccf-mtDNA levels did not differ significantly between PTSD subjects with and without concurrent MDD, including when controlling for age, diabetes status, and antidepressant use, suggesting that the association we observed between PTSD status and ccf-mtDNA was independent of MDD status. On the other hand, antidepressant users within the PTSD group had significantly higher ccf-mtDNA levels when controlling for age and diabetes status. This finding raising the possibility of a positive association between antidepressant use, itself, and increased ccf-mtDNA levels within the PTSD group may be consistent with prior work showing direct effects of antidepressants on mitochondrial function and integrity [53–56]. Antidepressants’ effects on mitochondrial function may include inhibition of key enzymes in the mitochondrial respiratory chain [53], which could lead to apoptosis and thus ccf-mtDNA release. While several studies have reported that antidepressants may exert pro-apoptotic effects, others have reported anti-apoptotic effects [57]. One animal study [58] suggested that pro- or anti-apoptotic effects may be dependent on the psychological state of the subject receiving the antidepressant. In a rodent model of chronic social isolation stress, this study found that SSRI treatment was associated with several hallmarks of apoptosis, but this was more pronounced in stressed animals than in non-stressed animals [58]. This suggests that there may be synergistic effects at play in stressed individuals such as oxidative stress or HPA-axis hyperactivity that could lead to apoptosis following antidepressant use, which may have contributed to an apparent antidepressant-associated increase in ccf-mtDNA levels in the PTSD group. While our data do not offer any clues as to causality, they do suggest that antidepressant use should be considered a relevant variable in mitochondrial studies.

PTSD has been associated with an increased risk of developing Type 2 diabetes [36, 59], and elevated HbA1c was reported to be a risk factor for developing PTSD [60]. Type 2 diabetes is associated with elevated ccf-mtDNA levels [5–9], and HbA1c has been positively correlated with ccf-mtDNA levels in diabetic patients [7]. There was a higher number of subjects who met criteria for diabetes in the PTSD positive group than in the PTSD negative group, though the difference missed significance. Because of the increased prevalence of glucose dysregulation in PTSD, the results from our sensitivity and correlation analyses suggest that future studies of ccf-mtDNA should also take diabetes status and/or HbA1c levels into consideration.

The results of our analyses of glucocorticoid sensitivity add support to the growing evidence that glucocorticoids and ccf-mtDNA may be related. Previous studies reported positive correlations between ccf-mtDNA levels and post-dexamethasone cortisol [22] and salivary cortisol following exercise [23]. PTSD is associated with a hypersensitive negative feedback response in the HPA axis, and previous findings have suggested it could be related to increased glucocorticoid receptor (GR) sensitivity [50, 61, 62], which may contribute to the development of PTSD [2]. However, it has also been hypothesized that the increased responsiveness to glucocorticoids is instead a product of trauma exposure [63, 64]. Increased dexamethasone-induced suppression of cortisol [56, 57] and ACTH [58, 59] were reported in previous studies of PTSD. Consistent with this, we found significantly elevated glucocorticoid sensitivity in PBMCs from the PTSD group. Although the precise relationship between glucocorticoids and ccf-mtDNA levels remains unclear, our data are consistent with growing evidence of the importance of glucocorticoid interactions with mitochondria [65–68]. Based on our findings, investigating the associations between ccf-mtDNA and specific molecules involved in GR signaling would be of interest. GRs enter mitochondria and directly interact with mitochondrial DNA [69–72], and molecules associated with GR entry and activity within mitochondria, such as Bag-1 [73], Bcl-2 [66], FKBP51 [74], HDAC6 [75], and Hsp90 [73], could be interesting candidates.

Several studies have suggested that acute psychological stress leads to an increase in ccf-mtDNA [4, 23, 24], though the exact upstream mechanisms triggering ccf-mtDNA release in these cases are not fully understood. A study using cultured human fibroblasts found that glucocorticoid administration can trigger extrusion of mtDNA into the cytosol [22], and oxidative stress, which can be induced by glucocorticoids [76], has also been shown to induce the release of cytosolic mtDNA [77], possibly via pores assembled by the protein voltage-dependent anion channel located on the outer mitochondrial membrane [78]. Ccf-mtDNA can be released passively via different forms of cell death or actively via regulated processes [4], and a clearer understanding of the mechanisms governing the release of cytosolic mtDNA from intact cells seems important for shedding light on the nature of the relationship between psychological stress, glucocorticoids, and ccf-mtDNA levels.

Lower ccf-mtDNA levels have been found in the CSF of subjects in the early stages of Parkinson’s and Alzheimer’s disease [16, 17]. In both cases, the mechanisms underlying the lower CSF ccf-mtDNA levels are not currently understood, but it was hypothesized that the reduction in ccf-mtDNA in those cases could accompany a decline in mtDNA resulting from mitochondrial dysfunction that occurs prior to cell death [16–18]. Alternatively, pathologically decreased ccf-mtDNA levels could be a consequence of the drive to increase cellular mtDNA content by restricting the fraction of mtDNA that is released [79]. PTSD has been associated with an increased risk of neurodegenerative disorders [80], and mitochondrial dysfunction has been implicated in PTSD [35, 81–83]. Kageyama et al. [84] reported that transgenic mice whose forebrain neurons expressed a mutant form of *Plog1*, which results in an increase in mtDNA deletions, had a significantly lower C01/D-loop ratio in their plasma ccf-mtDNA relative to controls, suggesting brain-derived mtDNA can enter the plasma. If this is the case in humans, it is plausible that changes in mitochondrial function in the brain could have contributed to the differences observed here, though further research would be required to determine the impact of brain-derived ccf-mtDNA on plasma ccf-mtDNA levels. Importantly, the degree to which peripheral measures of ccf-mtDNA levels reflect central vs. other sources is unknown, and nothing in our data directly implicates central processes.

The present study has several strengths. We recruited a relatively large, well-characterized sample of young and healthy participants, and our exclusion criteria reduced the likelihood that biochemical measurements were influenced by other medical comorbidities. Moreover, because the PTSD and control groups had all experienced combat trauma, we were able to control for the possibility that the differences observed were due to trauma experience itself. On the other hand, however, since the PTSD-negative controls had experienced significant combat trauma, they may have represented an especially resilient group of individuals. The relatively large size of our cohort relative to most other ccf-mtDNA studies to date increased the statistical power of our correlations, making this a significant contribution to the current ccf-mtDNA literature. Our replication of the positive associations between ccf-mtDNA levels and age and HbA1c suggests that these associations may be stable across various populations and should be considered in future studies of ccf-mtDNA levels. Finally, while there have been studies of ccf-mtDNA levels in other psychiatric disorders, this is the first study to investigate ccf-mtDNA levels in PTSD. Limitations to our study include having only male combat veterans. As a result, it is unclear whether our findings are generalizable to civilians or to females with PTSD. In addition, because studies thus far have had considerable variation in the blood fraction used and DNA purification protocols, our results can only be directly compared with studies using similar protocols. Moreover, ccf-mtDNA levels were only measured once in this study. Because there can be substantial variations in ccf-mtDNA levels within individual subjects over time and depending on psychological state [4, 85] and physical activity [4, 23, 26, 86, 87], measurements at multiple time points along with a stress assessment and a record of physical activity at each blood draw would be ideal. In addition, although our exclusionary criteria included a history of moderate to severe traumatic brain injury, we did not collect data on injury characteristics in participants whose trauma involved physical injury. Increases in ccf-mtDNA have been reported in studies of hospital patients admitted for traumatic brain injury [88], hip fracture [89], blunt trauma [90–92], and trauma requiring ICU admission [93]. Of the two studies that took multiple measurements over a 5-day period, both reported significantly elevated ccf-mtDNA levels in patients on day 1 [91, 92, 94]. However, while one study found ccf-mtDNA levels remained significantly higher than healthy controls at all time points [92, 94], the other reported a significant decline following day 1 [91, 94]. In our study, ccf-mtDNA levels were measured over 6 years, on average, after index trauma, and further research is required to determine if acute injury has lasting effects on ccf-mtDNA levels. Moreover, though there were significantly more smokers in the PTSD group, many subjects were missing smoking data, so smoking status was not included in our analyses. Although the precise relationship between ccf-mtDNA and smoking is not yet clear, the limited number of available studies [95, 96] suggest smoking may increase ccf-mtDNA, and this may have impacted ccf-mtDNA levels in our cohort. The influence of smoking, race/ethnicity, and other possible confounding variables on ccf-mtDNA levels should be assessed further in future studies. Another potential limitation to interpreting our results is that the primer set we used for amplifying mitochondrial DNA targeted a sequence that is also present in a nuclear mitochondrial pseudogene (NUMT) on chromosome 1. However, it does not seem likely that this significantly impacted our ccf-mtDNA data for the following reasons. We had attempted to amplify any nuclear DNA present our plasma samples using a Taqman assay (Thermo Fisher Scientific, Cat# 4403326) with RNase P as the target, but it did not yield any PCR product, suggesting that there was no detectable nuclear genome in the DNA from the plasma samples. Moreover, Meddeb et al. showed that in the plasma of healthy individuals, there are approximately 50,000 times more copies of ccf-mtDNA than circulating cell-free nuclear DNA (ccf-nDNA) [97]. Given the low abundance of ccf-nDNA in plasma, even in the case that nuclear DNA had been present in our plasma samples, this could have elevated the levels of ccf-mtDNA in theory but likely not to a magnitude that would bias the results. Nevertheless, targeting mitochondrial sequences that are not shared by any NUMTs would be an important consideration for future assays of ccf-mtDNA levels. Finally, ccf-mtDNA comprises not only non-membrane bound DNA fragments but also mtDNA contained in cell-free intact mitochondria and in extracellular vesicles (EV) such as microvesicles and exosomes, each with potentially distinct mechanisms of release and physiological roles, and different purification protocols result in discrepancies in the type of ccf-mtDNA isolated [4, 98], potentially impacting results. Based on our protocol, our samples should have contained non-membrane bound ccf-mtDNA in addition to that contained in microvesicles and exosomes [50], and we found a clear positive correlation with aging. On the other hand, Lazo et al. [99] analyzed only EV-bound ccf-mtDNA and found a negative association. Future investigations of PTSD’s effect on each type of ccf-mtDNA separately would help clarify the clinical significance of our findings.

In conclusion, using a relatively large, well-phenotyped veteran male sample, we found no overall between-group difference in ccf-mtDNA levels in unadjusted analyses. After controlling for age, diabetes status, and antidepressant use, however, those with PTSD showed lower ccf-mtDNA levels than those without PTSD. Thus, while PTSD per se is not associated with altered plasma ccf-mtDNA levels, a subgroup of individuals with PTSD who do not have diabetes or take antidepressants may show decreased levels. Our results also suggest that elevated glucocorticoid sensitivity may be associated with lower ccf-mtDNA levels. This observation may tie together the increased glucocorticoid sensitivity reported in PTSD with our observation of decreased ccf-mtDNA, at least in this subgroup. Finally, our results are consistent with literature suggesting mitochondrial involvement in PTSD [35, 81–83], at least in individuals who are not diabetic or taking antidepressant medication, although the pathophysiological significance of low plasma ccf-mtDNA levels remains uncertain. Although ccf-mtDNA’s usefulness as a diagnostic biomarker of PTSD is doubtful, our results suggest that improving our understanding of ccf-mtDNA in PTSD could aid in elucidating the mechanisms underlying PTSD’s pathophysiology and the relationships between glucocorticoid signaling, antidepressants, and mitochondrial function.

## Data Availability

The data that support the findings of this study are available from the corresponding author, GW, upon reasonable request.

## References

[1] Karam EG, Friedman MJ, Hill ED, Kessler RC, McLaughlin KA, Petukhova M (2014). Cumulative traumas and risk thresholds: 12-month PTSD in the World Mental Health (WMH) surveys. Depress Anxiety.

[2] Yehuda R, Hoge CW, McFarlane AC, Vermetten E, Lanius RA, Nievergelt CM (2015). Post-traumatic stress disorder. Nat Rev Dis Prim.

[3] Dursa EK, Reinhard MJ, Barth SK, Schneiderman AI (2014). Prevalence of a positive screen for PTSD among OEF/OIF and OEF/OIF-era veterans in a large population-based cohort. J Trauma Stress.

[4] Trumpff C, Michelson J, Lagranha CJ, Taleon V, Karan KR, Sturm G (2021). Stress and circulating cell-free mitochondrial DNA: a systematic review of human studies, physiological considerations, and technical recommendations. Mitochondrion.

[5] Liu J, Cai X, Xie L, Tang Y, Cheng J, Wang J (2015). Circulating cell free mitochondrial DNA is a biomarker in the development of coronary heart disease in the patients with type 2 diabetes. Clin Lab.

[6] Liu J, Zou Y, Tang Y, Xi M, Xie L, Zhang Q (2016). Circulating cell-free mitochondrial deoxyribonucleic acid is increased in coronary heart disease patients with diabetes mellitus. J Diabetes Investig.

[7] Bae JH, Jo SI, Kim SJ, Lee JM, Jeong JH, Kang JS, et al. Circulating cell-free mtDNA contributes to AIM2 inflammasome-mediated chronic inflammation in patients with type 2 diabetes. Cells. 2019;8:328. 10.3390/cells8040328 PMC6524162 30965677

[8] Silzer T, Barber R, Sun J, Pathak G, Johnson L, O’Bryant S (2019). Circulating mitochondrial DNA: New indices of type 2 diabetes-related cognitive impairment in Mexican Americans. PLoS ONE.

[9] Yuzefovych LV, Pastukh VM, Ruchko MV, Simmons JD, Richards WO, Rachek LI (2019). Plasma mitochondrial DNA is elevated in obese type 2 diabetes mellitus patients and correlates positively with insulin resistance. PLoS ONE.

[10] Boyapati RK, Tamborska A, Dorward DA, Ho GT (2017). Advances in the understanding of mitochondrial DNA as a pathogenic factor in inflammatory diseases. F1000Res.

[11] Pinti M, Cevenini E, Nasi M, De Biasi S, Salvioli S, Monti D (2014). Circulating mitochondrial DNA increases with age and is a familiar trait: Implications for “inflamm-aging. Eur J Immunol.

[12] Padilla-Sánchez SD, Navarrete D, Caicedo A, Teran E (2020). Circulating cell-free mitochondrial DNA levels correlate with body mass index and age. Biochim Biophys Acta Mol Basis Dis.

[13] Schwarzenbach H, Hoon DS, Pantel K (2011). Cell-free nucleic acids as biomarkers in cancer patients. Nat Rev Cancer.

[14] Li L, Hann HW, Wan S, Hann RS, Wang C, Lai Y (2016). Cell-free circulating mitochondrial DNA content and risk of hepatocellular carcinoma in patients with chronic HBV infection. Sci Rep.

[15] Meng X, Schwarzenbach H, Yang Y, Müller V, Li N, Tian D (2019). Circulating mitochondrial DNA is linked to progression and prognosis of epithelial ovarian cancer. Transl Oncol.

[16] Podlesniy P, Figueiro-Silva J, Llado A, Antonell A, Sanchez-Valle R, Alcolea D (2013). Low cerebrospinal fluid concentration of mitochondrial DNA in preclinical Alzheimer disease. Ann Neurol.

[17] Pyle A, Brennan R, Kurzawa-Akanbi M, Yarnall A, Thouin A, Mollenhauer B (2015). Reduced cerebrospinal fluid mitochondrial DNA is a biomarker for early-stage Parkinson’s disease. Ann Neurol.

[18] Gambardella S, Limanaqi F, Ferese R, Biagioni F, Campopiano R, Centonze D (2019). ccf-mtDNA as a potential link between the brain and immune system in neuro-immunological disorders. Front Immunol.

[19] Gonçalves VF, Mendes-Silva AP, Koyama E, Vieira E, Kennedy JL, Diniz B (2021). Increased levels of circulating cell-free mtDNA in plasma of late life depression subjects. J Psychiatr Res.

[20] Ampo E, Mendes-Silva AP, Goncalves V, Bartley JM, Kuchel GA, Diniz BS (2022). Increased levels of circulating cell-free mtDNA in the plasma of subjects with late-life depression and frailty: a preliminary study. Am J Geriatr Psychiatry.

[21] Lindqvist D, Wolkowitz OM, Picard M, Ohlsson L, Bersani FS, Fernström J (2018). Circulating cell-free mitochondrial DNA, but not leukocyte mitochondrial DNA copy number, is elevated in major depressive disorder. Neuropsychopharmacology.

[22] Lindqvist D, Fernström J, Grudet C, Ljunggren L, Träskman-Bendz L, Ohlsson L (2016). Increased plasma levels of circulating cell-free mitochondrial DNA in suicide attempters: associations with HPA-axis hyperactivity. Transl Psychiatry.

[23] Hummel EM, Hessas E, Müller S, Beiter T, Fisch M, Eibl A (2018). Cell-free DNA release under psychosocial and physical stress conditions. Transl Psychiatry.

[24] Trumpff C, Marsland AL, Basualto-Alarcón C, Martin JL, Carroll JE, Sturm G (2019). Acute psychological stress increases serum circulating cell-free mitochondrial DNA. Psychoneuroendocrinology.

[25] Kageyama Y, Kasahara T, Kato M, Sakai S, Deguchi Y, Tani M (2018). The relationship between circulating mitochondrial DNA and inflammatory cytokines in patients with major depression. J Affect Disord.

[26] Fernström J, Ohlsson L, Asp M, Lavant E, Holck A, Grudet C (2021). Plasma circulating cell-free mitochondrial DNA in depressive disorders. PLoS ONE.

[27] Lindqvist D, Furmark T, Lavebratt C, Ohlsson L, Månsson KNT (2023). Plasma circulating cell-free mitochondrial DNA in social anxiety disorder. Psychoneuroendocrinology.

[28] Behnke A, Gumpp AM, Rojas R, Sänger T, Lutz-Bonengel S, Moser D (2023). Circulating inflammatory markers, cell-free mitochondrial DNA, cortisol, endocannabinoids, and N-acylethanolamines in female depressed outpatients. World J Biol Psychiatry.

[29] Stertz L, Fries GR, Rosa AR, Kauer-Sant’anna M, Ferrari P, Paz AV (2015). Damage-associated molecular patterns and immune activation in bipolar disorder. Acta Psychiatr Scand.

[30] Jeong H, Dimick MK, Sultan A, Duong A, Park SS, El Soufi El Sabbagh D (2020). Peripheral biomarkers of mitochondrial dysfunction in adolescents with bipolar disorder. J Psychiatr Res.

[31] Park SS, Jeong H, Andreazza AC (2022). Circulating cell-free mitochondrial DNA in brain health and disease: a systematic review and meta-analysis. World J Biol Psychiatry.

[32] Panagioti M, Gooding P, Tarrier N (2009). Post-traumatic stress disorder and suicidal behavior: a narrative review. Clin Psychol Rev.

[33] Wilcox HC, Storr CL, Breslau N (2009). Posttraumatic stress disorder and suicide attempts in a community sample of urban american young adults. Arch Gen Psychiatry.

[34] Pompili M, Sher L, Serafini G, Forte A, Innamorati M, Dominici G (2013). Posttraumatic stress disorder and suicide risk among veterans: a literature review. J Nerv Ment Dis.

[35] Mellon SH, Gautam A, Hammamieh R, Jett M, Wolkowitz OM (2018). Metabolism, metabolomics, and inflammation in posttraumatic stress disorder. Biol Psychiatry.

[36] Boyko EJ, Jacobson IG, Smith B, Ryan MA, Hooper TI, Amoroso PJ (2010). Risk of diabetes in U.S. military service members in relation to combat deployment and mental health. Diabetes Care.

[37] Rohleder N, Joksimovic L, Wolf JM, Kirschbaum C (2004). Hypocortisolism and increased glucocorticoid sensitivity of pro-Inflammatory cytokine production in Bosnian war refugees with posttraumatic stress disorder. Biol Psychiatry.

[38] de Kloet CS, Vermetten E, Geuze E, Kavelaars A, Heijnen CJ, Westenberg HG (2006). Assessment of HPA-axis function in posttraumatic stress disorder: pharmacological and non-pharmacological challenge tests, a review. J Psychiatr Res.

[39] Lindqvist D, Wolkowitz OM, Mellon S, Yehuda R, Flory JD, Henn-Haase C (2014). Proinflammatory milieu in combat-related PTSD is independent of depression and early life stress. Brain Behav Immun.

[40] Hammamieh R, Chakraborty N, Gautam A, Muhie S, Yang R, Donohue D (2017). Whole-genome DNA methylation status associated with clinical PTSD measures of OIF/OEF veterans. Transl Psychiatry.

[41] Dean KR, Hammamieh R, Mellon SH, Abu-Amara D, Flory JD, Guffanti G (2020). Multi-omic biomarker identification and validation for diagnosing warzone-related post-traumatic stress disorder. Mol Psychiatry.

[42] Wisco BE, Marx BP, Wolf EJ, Miller MW, Southwick SM, Pietrzak RH (2014). Posttraumatic stress disorder in the US veteran population: results from the National Health and Resilience in Veterans Study. J Clin Psychiatry.

[43] Blake DD, Weathers FW, Nagy LM, Kaloupek DG, Gusman FD, Charney DS (1995). The development of a Clinician-Administered PTSD Scale. J Trauma Stress.

[44] Weathers FW, Keane TM, Davidson JR (2001). Clinician-administered PTSD scale: a review of the first ten years of research. Depress Anxiety.

[45] Corrigan JD, Bogner J (2007). Initial reliability and validity of the Ohio State University TBI Identification Method. J Head Trauma Rehabil.

[46] First MB, Gibbon M. *The Structured Clinical Interview For DSM-IV Axis I Disorders (SCID-I) And The Structured Clinical Interview For DSM-IV Axis II Disorders (SCID-II)* (John Wiley & Sons, Inc., 2004).

[47] Blanchard EB, Jones-Alexander J, Buckley TC, Forneris CA (1996). Psychometric properties of the PTSD Checklist (PCL). Behav Res Ther.

[48] Beck AT, Steer RA, Ball R, Ranieri W (1996). Comparison of beck depression inventories -IA and -II in psychiatric outpatients. J Pers Assess.

[49] Cohen S, Kamarck T, Mermelstein R (1983). A global measure of perceived stress. J Health Soc Behav.

[50] Yehuda R, Golier JA, Yang RK, Tischler L (2004). Enhanced sensitivity to glucocorticoids in peripheral mononuclear leukocytes in posttraumatic stress disorder. Biol Psychiatry.

[51] Somvanshi PR, Mellon SH, Yehuda R, Flory JD, Makotkine I, Bierer L (2020). Role of enhanced glucocorticoid receptor sensitivity in inflammation in PTSD: insights from computational model for circadian-neuroendocrine-immune interactions. Am J Physiol Endocrinol Metab.

[52] Blom G. *Statistical Estimates and Transformed Beta-Variables* (John Wiley & Sons, 1958)

[53] Abdel-Razaq W, Kendall DA, Bates TE (2011). The effects of antidepressants on mitochondrial function in a model cell system and isolated mitochondria. Neurochem Res.

[54] Then CK, Liu KH, Liao MH, Chung KH, Wang JY, Shen SC (2017). Antidepressants, sertraline and paroxetine, increase calcium influx and induce mitochondrial damage-mediated apoptosis of astrocytes. Oncotarget.

[55] Allen J, Romay-Tallon R, Brymer KJ, Caruncho HJ, Kalynchuk LE (2018). Mitochondria and mood: mitochondrial dysfunction as a key player in the manifestation of depression. Front Neurosci.

[56] Turck CW, Webhofer C, Reckow S, Moy J, Wang M, Guillermier C (2022). Antidepressant treatment effects on hippocampal protein turnover: molecular and spatial insights from mass spectrometry. Proteomics.

[57] Caiaffo V, Oliveira BD, de Sá FB, Evêncio Neto J (2016). Anti-inflammatory, antiapoptotic, and antioxidant activity of fluoxetine. Pharm Res Perspect.

[58] Djordjevic J, Djordjevic A, Adzic M, Elaković I, Matić G, Radojcic MB (2011). Fluoxetine affects antioxidant system and promotes apoptotic signaling in Wistar rat liver. Eur J Pharmacol.

[59] Scherrer JF, Salas J, Norman SB, Schnurr PP, Chard KM, Tuerk P (2019). Association between clinically meaningful posttraumatic stress disorder improvement and risk of type 2 diabetes. JAMA Psychiatry.

[60] Gandubert C, Scali J, Ancelin ML, Carrière I, Dupuy AM, Bagnolini G (2016). Biological and psychological predictors of posttraumatic stress disorder onset and chronicity. A one-year prospective study. Neurobiol Stress.

[61] Yehuda R, Boisoneau D, Lowy MT, Giller EL (1995). Dose-response changes in plasma cortisol and lymphocyte glucocorticoid receptors following dexamethasone administration in combat veterans with and without posttraumatic stress disorder. Arch Gen Psychiatry.

[62] Yehuda R, Yang RK, Golier JA, Grossman RA, Bierer LM, Tischler L (2006). Effect of sertraline on glucocorticoid sensitivity of mononuclear leukocytes in post-traumatic stress disorder. Neuropsychopharmacology.

[63] Daskalakis NP, Lehrner A, Yehuda R (2013). Endocrine aspects of post-traumatic stress disorder and implications for diagnosis and treatment. Endocrinol Metab Clin North Am.

[64] de Voogd LD, Kampen RA, Kaldewaij R, Zhang W, Hashemi MM, Koch SBJ (2022). Trauma-induced human glucocorticoid receptor expression increases predict subsequent HPA-axis blunting in a prospective longitudinal design. Psychoneuroendocrinology.

[65] Du J, McEwen B, Manji HK (2009). Glucocorticoid receptors modulate mitochondrial function: a novel mechanism for neuroprotection. Commun Integr Biol.

[66] Du J, Wang Y, Hunter R, Wei Y, Blumenthal R, Falke C (2009). Dynamic regulation of mitochondrial function by glucocorticoids. Proc Natl Acad Sci USA.

[67] Picard M, McEwen BS, Epel ES, Sandi C (2018). An energetic view of stress: Focus on mitochondria. Front Neuroendocrinol.

[68] Choi GE, Han HJ (2021). Glucocorticoid impairs mitochondrial quality control in neurons. Neurobiol Dis.

[69] Psarra AM, Sekeris CE (2011). Glucocorticoids induce mitochondrial gene transcription in HepG2 cells: role of the mitochondrial glucocorticoid receptor. Biochim Biophys Acta.

[70] Lee SR, Kim HK, Song IS, Youm J, Dizon LA, Jeong SH (2013). Glucocorticoids and their receptors: insights into specific roles in mitochondria. Prog Biophys Mol Biol.

[71] Lapp HE, Bartlett AA, Hunter RG (2019). Stress and glucocorticoid receptor regulation of mitochondrial gene expression. J Mol Endocrinol.

[72] Kokkinopoulou I, Moutsatsou P. Mitochondrial glucocorticoid receptors and their actions. Int J Mol Sci. 2021;22:6054. 10.3390/ijms22116054 PMC8200016 34205227

[73] Luo S, Hou Y, Zhang Y, Feng L, Hunter RG, Yuan P (2021). Bag-1 mediates glucocorticoid receptor trafficking to mitochondria after corticosterone stimulation: Potential role in regulating affective resilience. J Neurochem.

[74] Gallo LI, Lagadari M, Piwien-Pilipuk G, Galigniana MD (2011). The 90-kDa heat-shock protein (Hsp90)-binding immunophilin FKBP51 is a mitochondrial protein that translocates to the nucleus to protect cells against oxidative stress. J Biol Chem.

[75] Li ZY, Li QZ, Chen L, Chen BD, Zhang C, Wang X (2016). HPOB, an HDAC6 inhibitor, attenuates corticosterone-induced injury in rat adrenal pheochromocytoma PC12 cells by inhibiting mitochondrial GR translocation and the intrinsic apoptosis pathway. Neurochem Int.

[76] Costantini D, Marasco V, Møller AP (2011). A meta-analysis of glucocorticoids as modulators of oxidative stress in vertebrates. J Comp Physiol B.

[77] Nakahira K, Haspel JA, Rathinam VA, Lee SJ, Dolinay T, Lam HC (2011). Autophagy proteins regulate innate immune responses by inhibiting the release of mitochondrial DNA mediated by the NALP3 inflammasome. Nat Immunol.

[78] Kim J, Gupta R, Blanco LP, Yang S, Shteinfer-Kuzmine A, Wang K (2019). VDAC oligomers form mitochondrial pores to release mtDNA fragments and promote lupus-like disease. Science.

[79] Lowes H, Pyle A, Santibanez-Koref M, Hudson G (2020). Circulating cell-free mitochondrial DNA levels in Parkinson’s disease are influenced by treatment. Mol Neurodegener.

[80] Song H, Sieurin J, Wirdefeldt K, Pedersen NL, Almqvist C, Larsson H (2020). Association of stress-related disorders with subsequent neurodegenerative diseases. JAMA Neurol.

[81] Su YA, Wu J, Zhang L, Zhang Q, Su DM, He P (2008). Dysregulated mitochondrial genes and networks with drug targets in postmortem brain of patients with posttraumatic stress disorder (PTSD) revealed by human mitochondria-focused cDNA microarrays. Int J Biol Sci.

[82] Zhang L, Li H, Hu X, Benedek DM, Fullerton CS, Forsten RD (2015). Mitochondria-focused gene expression profile reveals common pathways and CPT1B dysregulation in both rodent stress model and human subjects with PTSD. Transl Psychiatry.

[83] Bersani FS, Morley C, Lindqvist D, Epel ES, Picard M, Yehuda R (2016). Mitochondrial DNA copy number is reduced in male combat veterans with PTSD. Prog Neuropsychopharmacol Biol Psychiatry.

[84] Kageyama Y, Deguchi Y, Kasahara T, Tani M, Kuroda K, Inoue K (2022). Intra-individual state-dependent comparison of plasma mitochondrial DNA copy number and IL-6 levels in patients with bipolar disorder. J Affect Disord.

[85] Trumpff C, Marsland AL, Sloan RP, Kaufman BA, Picard M (2019). Predictors of ccf-mtDNA reactivity to acute psychological stress identified using machine learning classifiers: a proof-of-concept. Psychoneuroendocrinology.

[86] Stawski R, Walczak K, Kosielski P, Meissner P, Budlewski T, Padula G (2017). Repeated bouts of exhaustive exercise increase circulating cell free nuclear and mitochondrial DNA without development of tolerance in healthy men. PLoS ONE.

[87] Ohlsson L, Hall A, Lindahl H, Danielsson R, Gustafsson A, Lavant E (2020). Increased level of circulating cell-free mitochondrial DNA due to a single bout of strenuous physical exercise. Eur J Appl Physiol.

[88] Marcatti M, Saada J, Okereke I, Wade CE, Bossmann SH, Motamedi M (2021). Quantification of circulating cell free mitochondrial DNA in extracellular vesicles with picoGreen™ in liquid biopsies: fast assessment of disease/trauma severity. Cells.

[89] Zhang JZ, Wang J, Qu WC, Wang XW, Liu Z, Ren JX (2017). Plasma mitochondrial DNA levels were independently associated with lung injury in elderly hip fracture patients. Injury.

[90] Lam NYL, Rainer TH, Chiu RWK, Joynt GM, Lo YMD (2004). Plasma mitochondrial DNA concentrations after trauma. Clin Chem.

[91] Yamanouchi S, Kudo D, Yamada M, Miyagawa N, Furukawa H, Kushimoto S (2013). Plasma mitochondrial DNA levels in patients with trauma and severe sepsis: time course and the association with clinical status. J Crit Care.

[92] McIlroy DJ, Bigland M, White AE, Hardy BM, Lott N, Smith DW (2015). Cell necrosis-independent sustained mitochondrial and nuclear DNA release following trauma surgery. J Trauma Acute Care Surg.

[93] Gu X, Yao Y, Wu G, Lv T, Luo L, Song Y (2013). The plasma mitochondrial DNA is an independent predictor for post-traumatic systemic inflammatory response syndrome. PLoS ONE.

[94] Thurairajah K, Briggs GD, Balogh ZJ (2018). The source of cell-free mitochondrial DNA in trauma and potential therapeutic strategies. Eur J Trauma Emerg Surg.

[95] Giordano L, Gregory AD, Pérez Verdaguer M, Ware SA, Harvey H, DeVallance E, et al. Extracellular release of mitochondrial dna: triggered by cigarette smoke and detected in COPD. Cells. 2022;11:369. 10.3390/cells11030369 PMC8834490 35159179

[96] Ueda K, Sakai C, Ishida T, Morita K, Kobayashi Y, Horikoshi Y (2023). Cigarette smoke induces mitochondrial DNA damage and activates cGAS-STING pathway: application to a biomarker for atherosclerosis. Clin Sci (Lond).

[97] Meddeb R, Dache ZAA, Thezenas S, Otandault A, Tanos R, Pastor B (2019). Quantifying circulating cell-free DNA in humans. Sci Rep.

[98] Al Amir Dache Z, Otandault A, Tanos R, Pastor B, Meddeb R, Sanchez C (2020). Blood contains circulating cell-free respiratory competent mitochondria. FASEB J.

[99] Lazo S, Noren Hooten N, Green J, Eitan E, Mode NA, Liu QR (2021). Mitochondrial DNA in extracellular vesicles declines with age. Aging Cell.

